# Diabetic Ketoacidosis–Associated Brain Injury: A Rare but Serious Complication

**DOI:** 10.1210/jcemcr/luaf103

**Published:** 2025-05-21

**Authors:** Bassam Bencharfa, Ilias Zegar, Sara Haddouga, Kenza Kehel, Ayoub Belhadj, Younes Aissaoui

**Affiliations:** Department of Critical Care Medicine, Avicenna Military Hospital, Marrakech 40000, Morocco; B2S Laboratory, Cadi Ayyad University, Faculty of Medicine and Pharmacy, Marrakech 40000, Morocco; Department of Critical Care Medicine, Avicenna Military Hospital, Marrakech 40000, Morocco; B2S Laboratory, Cadi Ayyad University, Faculty of Medicine and Pharmacy, Marrakech 40000, Morocco; Department of Critical Care Medicine, Avicenna Military Hospital, Marrakech 40000, Morocco; B2S Laboratory, Cadi Ayyad University, Faculty of Medicine and Pharmacy, Marrakech 40000, Morocco; Department of Critical Care Medicine, Avicenna Military Hospital, Marrakech 40000, Morocco; B2S Laboratory, Cadi Ayyad University, Faculty of Medicine and Pharmacy, Marrakech 40000, Morocco; Department of Critical Care Medicine, Avicenna Military Hospital, Marrakech 40000, Morocco; B2S Laboratory, Cadi Ayyad University, Faculty of Medicine and Pharmacy, Marrakech 40000, Morocco; Department of Critical Care Medicine, Avicenna Military Hospital, Marrakech 40000, Morocco; B2S Laboratory, Cadi Ayyad University, Faculty of Medicine and Pharmacy, Marrakech 40000, Morocco

**Keywords:** diabetic ketoacidosis, brain injury, seizure, cerebral edema, cerebral hypoperfusion, magnetic resonance imaging

## Abstract

Diabetic ketoacidosis (DKA)–associated brain injury is a rare but serious complication, typically occurring early during metabolic correction and associated with a poor prognosis. We report the case of an 18-year-old college student admitted to the intensive care unit with severe DKA, characterized by profound metabolic acidosis and marked electrolyte imbalances, including hypokalemia, hypernatremia, and hyperchloremia. Within hours, she developed altered mental status and generalized seizures. Brain magnetic resonance imaging (MRI) revealed diffuse cortical and brainstem abnormalities, suggestive of cytotoxic cerebral edema. She was managed with continuous sedation, mechanical ventilation, intravenous insulin, potassium supplementation, enteral nutrition, and gradual metabolic correction. Her neurologic status improved within a few days, and follow-up MRI showed partial regression of the lesions. She was subsequently transferred to the endocrinology department and ultimately regained full cognitive and physical function. This case underscores the importance of early recognition, close neurologic monitoring, timely neuroimaging, and tight metabolic and osmotic control to optimize outcomes in patients with DKA-associated brain injury.

## Introduction

Diabetic ketoacidosis (DKA)-associated brain injury is a rare but severe complication that typically occurs within the first few hours of metabolic correction. It is associated with poor outcomes and increased mortality [1]. The most serious neurological manifestation of DKA is cerebral edema (CE), which is well documented in pediatric populations, where it remains the leading cause of death, with a mortality rate of 20% to 25% [2, 3]. In contrast, CE is infrequently reported in adults. Its incidence, risk factors, and optimal management remain poorly defined [4].

Here, we present the case of a young patient with inaugural DKA and profound acidosis who subsequently developed DKA-associated brain injury. We describe the clinical presentation, imaging findings, pathophysiological hypotheses, and therapeutic strategies, emphasizing the importance of early recognition, close monitoring, and timely intervention in improving outcomes for this rare but potentially fatal complication.

## Case Presentation

An 18-year-old female college student with a history of chronic polydipsia and polyuria presented with a 1-week history of cough, fever, and pharyngitis. The day prior to admission, she developed recurrent vomiting. She was brought to the emergency department (ED) with altered mental status and respiratory distress.

On initial evaluation, she was tachycardic (130 beats per min), hypotensive (92/67 mm Hg), and tachypneic (35 breaths per min), with a capillary refill time of 3 seconds, sunken eyes, dry mucous membranes, and reduced urine output—findings consistent with both extracellular and intracellular dehydration. She was afebrile (36.1 °C), and neurologic assessment revealed a Glasgow Coma Scale (GCS) score of 13/15 (E3, V4, M6), with no focal neurologic deficits.

## Diagnostic Assessment

Point-of-care capillary glucose testing revealed severe hyperglycemia greater than 600 mg/dL (>33.3 mmol/L; normal range: 70-110 mg/dL [3.9-6.1 mmol/L]). Urine dipstick showed significant ketonuria, and a low venous bicarbonate level of 6 mmol/L (normal range: 22-30 mmol/L) confirmed the diagnosis of DKA [5].

Laboratory results revealed hypernatremia with a serum sodium of 143 mmol/L (normal reference range: 136-145 mmol/L) and a corrected sodium of 161 mmol/L. Additional abnormalities included profound hypokalemia, hyperchloremia, and severe metabolic acidosis. Inflammatory markers were elevated, with leukocytosis and thrombocytosis. Renal function was preserved, with a creatinine of 79 µmol/L (8.9 mg/dL) (normal range: 60-120 µmol/L; 5.7-10.9 mg/dL), and an estimated glomerular filtration rate of 82 mL/min/1.73 m^2^, calculated using the MDRD (Modification of Diet in Renal Disease) formula (reference: 95 ± 20 mL/min/1.73 m²).

## Treatment

Initial management in the ED included fluid resuscitation with 1 L of normal saline over the first hour. Following the initial bolus, fluid administration continued at 500 mL/h, titrated according to the hemodynamic response. The patient's water deficit was estimated to be approximately 3.7 L using the standard formula. Intravenous insulin therapy was initiated per local protocol, beginning with a 0.1 IU/kg bolus followed by a continuous infusion at 0.1 IU/kg/h.

Due to persistent altered mental status, severe metabolic acidosis, and profound hypokalemia (1.8 mmol/L; normal reference range: 3.5-4.6 mmol/L)—likely resulting from a combination of vomiting, osmotic diuresis, and insulin-induced intracellular potassium shift. The patient was transferred to the intensive care unit (ICU). On ICU admission, arterial blood gas analysis on room air revealed severe metabolic acidosis (pH 6.95, bicarbonate 5 mmol/L; normal reference range: pH 7.31-7.45, bicarbonate 22-33 mmol/L) with respiratory compensation (PaCO_2_ 27 mm Hg; normal reference range: 35-45 mmHg) (Table 1).

**Table 1.** Evolution of arterial blood gas, electrolytes, and renal function during intensive care unit stay

	H0	H12	H24	Day 3	Day 4	Day 9	Reference range
pH	**6.95**	**7.01**	**7.02**	**7.22**	7.32	**7.47**	7.31-7.45
PaCO_2_	**27 mm Hg** **(3.5 kPa)**	**25 mm Hg** **(3.3 kPa)**	**13 mm Hg** **(1.7 kPa)**	**21 mm Hg** **(2.7 kPa)**	**25 mm Hg** **(3.3 kPa)**	**34 mm Hg** **(4.5 kPa)**	35-45 mm Hg (4.7-6.0 kPa)
PaO_2_	90 mm Hg (11.9 kPa)	**132 mm Hg** **(17.5 kPa)**	**140 mm Hg** **(18.6 kPa)**	**114 mm Hg** **(15.1 kPa)**	**113 mm Hg** **(15.0 kPa)**	**160 mm Hg** **(21.2 kPa)**	80-100 mm Hg (10.7-13.3 kPa)
Bicarbonate	**5 mmol/L** **(5 meq/L)**	**6.3 mmol/L** **(6.3 meq/L)**	**5.4 mmol/L** **(5.4 meq/L)**	**11.4 mmol/L** **(11.4 meq/L)**	**15.6 mmol/L** **(15.6 meq/L)**	25.8 mmol/L (25.8 meq/L)	22-33 mmol/L (22-33 meq/L)
Base excess	**−25 mmol/L** **(−25 meq/L)**	**−23 mmol/L** **(−23 meq/L)**	**−25 mmol/L** **(−25 meq/L)**	**−17 mmol/L** **(−17 meq/L)**	**−13 mmol/L** **(−13 meq/L)**	1 mmol/L (1 meq/L)	−3.0 to +3.0 mmol/L −3.0 to +3.0 meq/L
Lactate	10.8 mg/dL (1.2 mmol/L)	3.6 mg/dL (0.4 mmol/L)	4.5 mg/dL (0.5 mmol/L)	6.3 mg/dL (0.7 mmol/L)	4.5 mg/dL (0.5 mmol/L)	9.0 mg/dL (1 mmol/L)	4.5-14.4 mg/dL (0.5-1.6 mmol/L)
Hemoglobin	14.5 g/dL		**11.7 g/dL**	**10.9 g/dL**	**8.9 g/dL**	**7.3 g/dL**	13-18 g/dL
Leukocytes	**15** **×** **10⁹/L**		**17.8** **×** **10⁹/L**	**25.8** **×** **10⁹/L**	**18.9** **×** **10⁹/L**	**13.9** **×** **10⁹/L**	4.00-11.00 × 10⁹/L
Platelets	169 × 10⁹/L		280 × 10⁹/L	264 × 10⁹/L	164 × 10⁹/L	269 × 10⁹/L	150-450 × 10⁹/L
Sodium	**153 mmol/L** **(153 meq/L)**	**161 mmol/L** **(161 meq/L)**	**161 mmol/L** **(161 meq/L)**	**160 mmol/L** **(160 meq/L)**	**156 mmol/L** **(156 meq/L)**	145 mmol/L (145 meq/L)	136-145 mmol/L (136-145 meq/L)
Potassium	**1.7 mmol/L** **(1.7 meq/L)**	**1.8 mmol/L** **(1.8 meq/L)**	**2.9 mmol/L** **(2.9 meq/L)**	4.4 mmol/L (4.4 meq/L)	**3 mmol/L** **(3 meq/L)**	4.4 mmol/L (4.4 meq/L)	3.5-4.6 mmol/L (3.5-4.6 meq/L)
Chloride	**126 mmol/L** **(126 meq/L)**		**140 mmol/L** **(140 meq/L)**	**130 mmol/L** **(130 meq/L)**	**124 mmol/L** **(124 meq/L)**		97-109 mmol/L (97-109 meq/L)
Glucose	**386 mg/dL** **(21.4 mmol/L)**	**209 mg/dL** **(11.6 mmol/L)**	**338 mg/dL** **(18.8 mmol/L)**	**179 mg/dL** **(9.9 mmol/L)**	**187 mg/dL** **(10.4 mmol/L)**	**181 mg/dL** **(10.1 mmol/L)**	<140 mg/dL (<7.8 mmol/L)
C-reactive protein	**8.2 mg/L**		**42 mg/L**	**54 mg/L**	**39 mg/L**	**33 mg/L**	<5 mg/L
Procalcitonin	**2.15 ng/mL**		**1.64 ng/mL**	**1.15 ng/mL**	0.4 ng/mL	0.3 ng/mL	<0.5 ng/mL
Creatinine	**13.1 mg/L** **(116 µmol/L)**		**15.5 mg/L** **(137 µmol/L)**	**26 mg/L** **(230 µmol/L)**	**26.6 mg/L** **(235 µmol/L)**	**18.4 mg/L** **(163 µmol/L)**	5.7-10.2 mg/L (60-120 µmol/L)
BUN	**11.6 mmol/L**		**14.2 mmol/L**	**15.3 mmol/L**	**15 mmol/L**	**17 mmol/L**	2.5-7.5 mmol/L
Urine ketones	**8 mmol/L**	**4 mmol/L**	**1.5 mmol/L**	<0.5 mmol/L	<0.5 mmol/L	<0.5 mmol/L	<0.5 mmol/L

Urine ketones were measured semi-quantitatively using Multistix reagent strips and converted to approximate absolute values (mmol/L) based on manufacturer data: negative (<0.5), trace (0.5), small (1.5), moderate (4.0), and large (8.0). Abnormal values shown in bold font. Values in parenthesis are International System of Units (SI).

Abbreviations: BUN, blood urea nitrogen; PaCO_2_, partial pressure of carbon dioxide in arterial blood (mm Hg-kPa); PaO₂, partial pressure of oxygen in arterial blood (mm Hg-kPa).

Fluid resuscitation continued in the ICU, guided by cardiac output monitoring using transthoracic echocardiography. Balanced crystalloid (Ringer lactate) was used. In total, the patient received 3.5 L of fluids over the first 36 hours—2 L of 0.9% saline in the ED and 1.5 L of Ringer lactate in the ICU. Insulin was withheld to avoid worsening hypokalemia. A jugular central venous catheter was placed, and intravenous potassium chloride was administered at 1 g/h. Once potassium levels partially corrected, insulin infusion was resumed at a reduced rate of 0.05 IU/kg/h. After resolution of ketosis, fluid therapy was adjusted to a maintenance rate of 30 mL/kg/day using either Ringer lactate or 5% dextrose, depending on glycemia. Enteral nutrition via a nasogastric tube was initiated on day 2 (500 kcal/day) and progressively increased to 1500 kcal/day by day 6.

A urinary tract infection (UTI) was suspected supported by a positive urine dipstick for nitrites and leukocytes. Empirical antibiotic therapy with ceftriaxone (2 g once daily) was initiated. Chest x-ray and abdominal ultrasound ruled out other infectious foci, and blood and urine cultures were obtained. Urinalysis showed a leukocyte count of 10⁴ cells/mL; however, urine culture grew fewer than 10³ colony-forming units, likely due to prior antibiotic exposure. A UTI was therefore identified as the most likely trigger for DKA.

**Figure 1.**
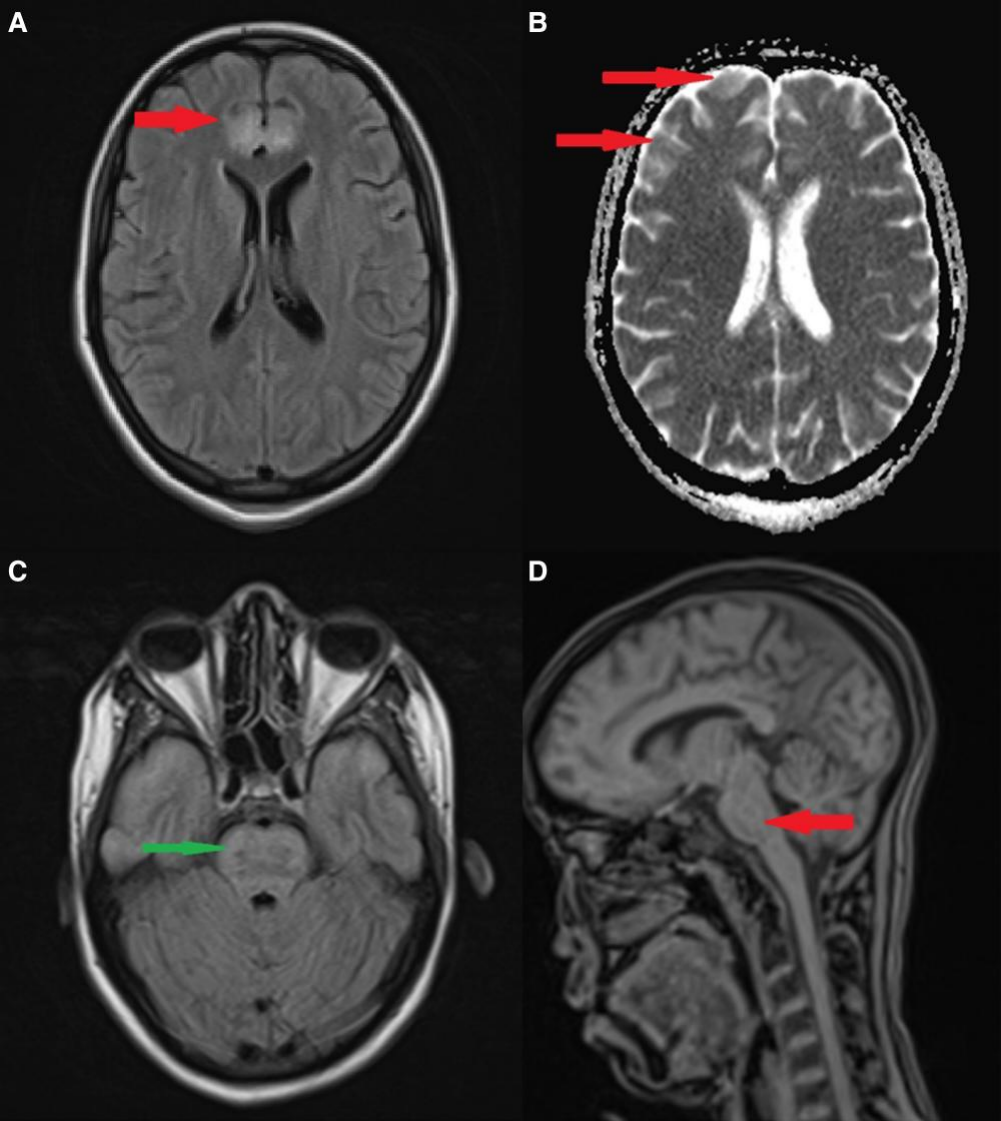
Magnetic resonance imaging (MRI) findings in a patient with diabetic ketoacidosis (DKA)-associated brain injury. A, Axial fluid-attenuated inversion recovery MRI showing cortical and subcortical hyperintensity in the left frontal lobe (arrow), suggestive of cerebral edema or ischemic injury. B, Axial diffusion-weighted imaging showing areas of diffusion restriction (arrows), consistent with cytotoxic edama and potential ischemic injury. C, Axial T1-weighted MRI demonstrating involvement of the brainstem (arrow), which may indicate hypoxic-ischemic injury or osmotic demyelination. D, The brainstem lesions are not enhanced after gadolinium injection. MRI findings were highly suggestive of ischemic pathway for brain damage. The reversible character as well as the rapid clinical recovery confirm the metabolic origin.

A few hours after ICU admission, the patient developed generalized tonic-clonic seizures. She was treated with intravenous midazolam (10 mg), followed by a phenobarbital loading dose (500 mg over 15 minutes). A brain computed tomography (CT) scan showed no acute abnormalities. Due to worsening neurologic status (GCS 8/15), endotracheal intubation and mechanical ventilation were initiated. Continuous sedation with midazolam was started, and propofol was added due to significant ventilator dyssynchrony. A lumbar puncture was also performed, revealing clear cerebrospinal fluid (CSF) with mildly elevated protein (1.2 g/L; normal reference range: <0.4 g/L), while glucose, chloride, and leukocyte counts remained within normal limits. CSF cultures were negative. Electroencephalography (EEG) demonstrated a flat tracing, likely attributable to deep sedation. Given the unexplained neurologic deterioration, brain magnetic resonance imaging (MRI) was performed and revealed multiple nonsystematized, symmetrical supratentorial lesions in the cortex and brainstem, consistent with cytotoxic cerebral edema of metabolic origin (Fig. 1).

During her ICU stay, she developed acute kidney injury, likely multifactorial in origin, related to hypovolemia, UTI, and hyperchloremia. The patient also experienced significant hypophosphatemia, attributed to insulin-driven intracellular shifts, osmotic diuresis, and possible respiratory alkalosis [6]. It was treated with intravenous phosphate. A progressive hemoglobin drop was noted in the ICU course, likely due to frequent blood draws, systemic inflammation, and possibly preexisting anemia masked by dehydration.

## Outcome and Follow-up

By day 5, the patient’s electrolyte imbalances had improved, and with metabolic stabilization, sedation was gradually discontinued, allowing for progressive recovery of consciousness. This enabled successful ventilator weaning and tracheal extubation by day 8.

A follow-up brain MRI showed persistent cortical and brainstem lesions, though with partial regression on diffusion-weighted imaging sequences. A repeat EEG demonstrated normal background activity and reactivity.

On day 11, the patient was transferred to the endocrinology department, where she was transitioned to subcutaneous insulin therapy. Acute kidney injury, which had begun to improve during her ICU stay, fully resolved during hospitalization. By day 20, she was discharged in stable condition without any neurological deficits.

## Discussion

This case highlights a reversible acute brain injury secondary to inaugural DKA. While well documented in pediatric populations, reports of DKA-associated brain injury in adults remain rare, underscoring the need for increased awareness and early recognition.

Classically, rapid fluid administration and subsequent shifts in serum osmolality were considered the primary cause of CE in DKA [7]. However, recent evidence indicates that cerebral hypoperfusion and the hyperinflammatory state induced by DKA play a central role in its pathogenesis [3].

According to the American Diabetes Association, fluid resuscitation in DKA should begin with 0.9% NaCl at 1 to 1.5 L over the first hour to restore intravascular volume [5]. Subsequent fluid choice (0.45% or 0.9% NaCl) depends on the corrected serum sodium. Although hypernatremia was present at admission, normal saline was used initially as it was the only available fluid in the ED. In the ICU, hypernatremia was corrected gradually to minimize the risk of osmotic cerebral injury, as rapid correction can lower plasma osmolality and trigger intracellular fluid shifts, potentially worsening cerebral edema.

The effect of intravenous fluid administration or abrupt glycemic correction on CE has been explored. A study by Kuppermann et al [8] assessed fluid administration rates and sodium chloride content in children with DKA, concluding that neither significantly influenced neurological outcomes. Similarly, a 2023 study [9] found that even with slow glycemic correction, some patients still developed CE.

While prospective studies on the pathophysiology of CE in DKA remain limited, Glaser et al [7] identified cerebral hypoperfusion and subsequent reperfusion as key contributors. Hypoperfusion results from reduced cerebral blood flow (CBF), exacerbated by profound hypocapnia. This process likely triggers ischemic injury, which may later progress to vasogenic edema due to increased CBF and blood-brain barrier dysfunction.

MRI studies provide further insights. One investigation assessed apparent diffusion coefficients (ADCs) as a marker of cerebral perfusion, performing MRIs 12 hours after DKA treatment initiation and 14 days later [10]. Findings suggested that vasogenic edema, rather than osmotic cellular swelling, was the predominant mechanism. However, in our case, an MRI performed 24 hours after treatment initiation revealed cytotoxic edema with low ADC values, indicating ischemic brain damage rather than vasogenic edema. It has been hypothesized that CBF increases beyond baseline levels following fluid resuscitation, leading to fluid extravasation and subsequent cytotoxic CE [11]. Consequently, we opted for a slow correction of hypocapnia to mitigate further neurological compromise.

Diffusion MRI revealed signal abnormalities in the frontal, temporal, and insular lobes, while the occipital lobe remained unaffected. These findings align with observational data describing cerebral alterations both in symptomatic and asymptomatic patients [12]. Additionally, ADC and perfusion ratios remained unchanged in posterior brain regions, possibly due to reduced sympathetic innervation and lower autoregulatory capacity of the vertebrobasilar system compared to the carotid circulation [13].

In pediatric DKA, CE has been linked to factors such as low PaCO_2_, elevated serum urea nitrogen, and bicarbonate administration [7]. In our patient, both low PaCO_2_ and increasing urea levels were observed. Severe hypocapnia persisted despite deep sedation, likely due to compensatory hyperventilation from metabolic acidosis, which may have aggravated cerebral hypoperfusion and ischemic injury. These findings support the hypothesis that acidosis-driven hyperventilation contributes to DKA-associated brain injury [14], emphasizing the need for close neurologic and ventilatory monitoring. While these risk factors are well studied in children, their applicability to adults remains uncertain. For instance, although age is not a significant risk factor in pediatric cohorts, Khan et al [4] identified advanced age as a predictor of CE in adults, suggesting potential differences in underlying pathophysiology.

Neurologic manifestations of DKA-associated brain injury typically include altered mental status, seizures, and signs of increased intracranial pressure [5, 7]. In our patient, after seizure onset, a comprehensive work-up was performed. Brain CT ruled out intracranial hypertension or hemorrhage, and lumbar puncture excluded central nervous system infection. MRI revealed symmetrical supratentorial lesions, which were probably responsible for the convulsions. In addition, profound metabolic acidosis and severe hypokalemia may have contributed by lowering the seizure threshold through increased neuronal excitability.

Management of DKA-associated brain injury centers on correcting metabolic abnormalities while minimizing factors that exacerbate cerebral edema. Gradual fluid resuscitation, careful glycemic control, and close monitoring of serum sodium are essential [8]. Electrolyte imbalances—especially hypokalemia and hypernatremia—should be corrected cautiously [5, 15]. In cases of neurologic deterioration, hyperosmolar therapy with hypertonic saline or mannitol may be considered, though adult data are limited [5, 16]. Mechanical ventilation with controlled CO_2_ levels can help prevent further cerebral hypoperfusion.

The prognosis of DKA-associated brain injury depends on the severity of cerebral edema, timeliness of intervention, and degree of metabolic derangement at presentation [7]. Outcomes range from full recovery to lasting neurologic deficits. In our case, the patient demonstrated significant clinical and radiological improvement, reinforcing the potential for recovery with appropriate supportive care. However, given the unpredictable nature of DKA-related neurological complications, long-term follow-up remains crucial.

DKA-associated brain injury is a rare but potentially life-threatening complication, often driven by cerebral hypoperfusion, ischemia-reperfusion injury, and blood-brain barrier dysfunction. While fluid and metabolic management remain the cornerstones of treatment, close neurological and ventilatory monitoring is essential, particularly in patients with persistent hypocapnia or severe metabolic acidosis. Given the limited data on DKA-related brain injury in adults, further research is needed to refine diagnostic strategies and preventive and therapeutic approaches to improve patient outcomes.

## Learning Points

- Early identification of DKA-associated brain injury and close neurological monitoring are crucial in DKA management to detect early signs of brain injury.
- DKA-associated brain injury is likely driven by ischemia-reperfusion mechanisms, leading to blood-brain barrier dysfunction and subsequent cerebral edema.
- MRI provides valuable insights into the underlying pathophysiology and helps rule out alternative diagnoses, though it may not always directly influence treatment decisions.
- Treatment is nonspecific and relies on progressive correction of metabolic disorders to prevent further neurological deterioration.

## Contributors

All authors made individual contributions to authorship. B.B., I.Z., K.K., S.H., A.B., and Y.A. were involved in the diagnosis and management of the patient and manuscript submission. B.B., I.Z., and Y.A. were the leads on the original draft. All authors reviewed and approved the final draft.

## Data Availability

Data sharing is not applicable to this article as no data sets were generated or analyzed during the current study.

## Abbreviations

ADCs: apparent diffusion coefficients
CBF: cerebral blood flow
CE: cerebral edema
CSF: cerebrospinal fluid
CT: computed tomography
DKA: diabetic ketoacidosis
ED: emergency department
EEG: electroencephalography
GCS: Glasgow Coma Scale
ICU: intensive care unit
MRI: magnetic resonance imaging
UTI: urinary tract infection
